# Single‐Cell Analysis of Endothelial Cell Injury in IgA Nephropathy

**DOI:** 10.1002/iid3.70149

**Published:** 2025-02-13

**Authors:** Yong‐Chang Yang, Lin Zhu, Jing‐Ying Zhao, Bo Zhang, Xiao‐Meng Wang, Wai W. Cheung, Cheng‐Guang Zhao, Ping Zhou

**Affiliations:** ^1^ Department of Pediatrics Shengjing Hospital of China Medical University Shenyang China; ^2^ Department of Pediatric Nephrology and Rheumatology Sichuan Provincial Women's and Children's Hospital/The Affiliated Women's and Children's Hospital of Chengdu Medical College Chengdu China; ^3^ Sichuan Clinical Research Center for Pediatric Nephrology Chengdu China; ^4^ Division of Pediatric Nephrology, Rady Children's Hospital University of California San Diego California USA; ^5^ Yangtze Delta Region Institute of Tsinghua University Jiaxing China

**Keywords:** endothelial cell, IgA nephropathy, IL‐6, Rac1, scRNA‐seq, VE‐cadherin

## Abstract

**Background:**

The precise mechanisms responsible for renal injury in IgA nephropathy (IgAN) are not fully understood. Our study employed an extensive scRNA‐seq analysis of kidney biopsies obtained from individuals with IgAN, with a specific emphasis on investigating the involvement of renal endothelial cells.

**Methods:**

We obtained data from the Gene Expression Omnibus database and conducted bioinformatics analysis, which included enrichment analysis of differentially expressed genes, AUCell analysis, and high‐dimensional weighted gene co‐expression network analysis (hdWGCNA). The results of these analyses were further validated using human renal glomerular endothelial cells (HRGECs).

**Results:**

The ScRNA‐seq data uncovered notable variations in gene expression between IgAN and control kidney tissues. The enrichment analysis using AUCell demonstrated a high presence of adhesion molecules and components related to the mitogen‐activated protein kinase signaling pathway within the renal endothelial cells. Furthermore, through hdWGCNA analysis, it was discovered that interleukin (IL)‐6, Rac1, and cadherin exhibited associations with the renal endothelial cells. Stimulation of HRGECs with IL‐6/IL‐6 receptor resulted in a significant reduction in VE‐cad expression while inhibiting Rac1 led to a substantial decrease in Rac1‐GTP levels and an increase in VE‐cad expression.

**Conclusion:**

This study presents novel findings regarding the contribution of renal endothelial cells to the development of IgAN, as it demonstrates that IL‐6 negatively regulates VE‐cad expression in HRGECs via Rac1. These results highlight the significant involvement of renal endothelial cells in the pathogenesis of IgAN.

**Abbreviations**
BP: biological processes
CAMs: cell adhesion molecules
CC: chemical processes
DEGs: differentially expressed genes
GEO: Gene Expression Omnibus
GO: gene ontogeny
hdWGCNA: high‐dimensional weighted gene co‐expression network analysis
HRGECs: human renal glomerular endothelial cells
IgAN: IgA nephropathy
IL: interleukin
KEGG: Kyoto Encyclopedia of Genes and Genomes
MAPK: mitogen‐activated protein kinase
MEs: modular signature genes
MF: molecular functions
RNA‐seq: RNA sequencing
scRNA‐seq: Single‐cell RNA sequencing
UMAP: unified manifold approximation and projection
WGCNA: weighted gene co‐expression network analysis

## 1 Introduction

Globally, IgA nephropathy (IgAN) is one of the most prevalent glomerular diseases. The likelihood of patients developing end‐stage renal disease within a span of 20–30 years after diagnosis is between 30% and 40% [1, 2]. In accordance with the multi‐hit hypothesis, IgAN is an autoimmune disease with immune pathogenesis [3]. Additionally, IgAN is influenced by the crosstalk between mesangial cells and podocytes, genetic factors, and complement activation [3]. Molecular pathways and cell types contributing to IgAN progression have not been comprehensively analyzed. There are many different types of specialized cells in the kidney, which are organized into functional zones. Due to this complexity, it is challenging to fully understand how kidney disease progresses and develops. Bulk RNA sequencing (RNA‐seq) is used to characterize transcriptional variations in various diseases. However, this method can only measure gene expression in many cells simultaneously [4]. In the total kidney cell population, specific kidney cell types that function abnormally may not be detected because it cannot distinguish between cell types. Single‐cell RNA sequencing (scRNA‐seq) is a novel approach to high‐throughput genome bioinformatics [5, 6]. By using scRNA‐seq, it is possible to determine a cell's type and gene expression status despite heterogeneity [7]. Recent studies have used this revolutionary approach to study kidney diseases pathogenesis [8]. A glomerular filtration barrier consists of the glomerular endothelium, basement membrane, and podocytes. Most studies of glomerular diseases focus on mesangial cells and podocytes; however, endothelial cell injury may also contribute to disease progression [9]. IgAN patients' glomerular endothelial cells are damaged in both pediatric and adult patients [10, 11]. We found that IL‐6 stimulates endothelial cell permeability in a rodent model of IgAN by downregulating cadherin, a crucial adhesion molecule [12]. The purpose of this study is to determine the pathogenesis of IgAN through the analysis of human scRNA‐seq data. To accomplish this, we performed bioinformatics analysis on the IgAN scRNA‐seq data set GSE171314 downloaded from the Gene Expression Omnibus (GEO) database. The results of the bioinformatics analyses were further verified on human glomerular endothelial cells.

## 2 Materials and Methods

### 2.1 Data Collection and Processing

From the NCBI GEO database, we obtained the scRNA‐seq data set GSE171314 [13]. Cells were harvested from four IgAN patients and one healthy control. We analyzed all scRNA‐seq data using the R package Seurat version 4.3.0 (https://satijalab.org/seurat/) [14].

### 2.2 scRNA‐Seq Quality Control and Integration

To obtain a reliable cell subpopulation, we first filtered single‐cell data with Seurat. We performed quality control according to the following criteria: (1) genes expressed in less than three cells; (2) cells expressing less than 200 genes; and (3) mitochondrial genes expressed at more than 40% intensity. To eliminate batch effects, cells from the four samples were integrated. The FindVariableFeatures function identified 2000 highly variable genes in the four samples. All genes were normalized and scaled with NorMalizeData and ScaleData to ensure data comparability. A cluster analysis was conducted using FindCluster (with a resolution of 0.3). We reduced gene expression matrices to 20 principal components and clustered them at 0.35 resolution through graph‐based clustering. ElBowPlot analysis was used to determine the appropriate principal component for dimensionality reduction. At a resolution of 0.3, cells were grouped into distinct clusters by unified manifold approximation and projection (UMAP) [15].

### 2.3 Cell Type‐Specific Marker Gene Analysis and Cell Annotation

Using the Wilks likelihood‐ratio test, the FindAllMarkers function in Seurat was used to determine the marker genes of each kidney cell population relative to the other cell clusters. More than 10% of the cells in a cluster expressed the selected marker genes, and the average log (multiple change) was greater than 0.25. CellMarker (http://117.50.127.228/CellMarker/) and established markers from the literature [16] were utilized to confirm the cell type (Table S1).

### 2.4 Identification of Differentially Expressed Genes

We identified differentially expressed genes (DEGs) in each kidney cell cluster by comparing gene transcription profiles between the IgAN and control groups. The Wilks likelihood‐ratio test was employed to assess the density disparity of individual cell clusters between the two groups, utilizing Seurat's FindMarkers function [17]. This gene's average expression across cell types is represented by Avg.exp. We considered DEGs with a *p* value of < 0.05 and an average log (multiple change) of > 0.25.

### 2.5 Enrichment Analysis

R package clusterProfiler version 3.16.1 was used to analyze gene ontogeny (GO) and Kyoto Encyclopedia of Genes and Genomes (KEGG) enrichment analyses. Enrichr (https://maayanlab.cloud/Enrichr/) was also used to analyze GO cellular components. GO terms were categorized into three groups: biological processes (BP), chemical processes (CC), and molecular functions (MF) [18]. In this study, *p* values were set at 0.05.

### 2.6 Protein Interaction Network Analysis

Using the STRING database version 11.0 (https://stringdb.org/), we evaluated protein interactions based on the first 500 DEGs [19]. To screen for significant interactions, we set a minimum interaction score of 0.9. We searched for hub genes with the Cytoscape plug‐in CytoHubba [20]. Using the bottleneck method, the ten most significant hub genes were identified.

### 2.7 AUCell Analysis

The AUC values of different pathway genes in different cells were calculated using the AUCell R package (Version 1.20.2). AUC values higher than one indicate a higher proportion of gene expression in the cell. Based on each cell's AUC score, the UMAP embedding is colored to show which cell clusters are active at the relevant gene concentrations.

### 2.8 High‐Dimensional Weighted Gene Co‐Expression Network Analysis

Biological systems are highly complex. To gain a better understanding of the specific gene expression patterns that are involved in multiple biological processes, weighted gene co‐expression network analysis (WGCNA) has been demonstrated to be an effective technique [21]. Traditional WGCNA, however, cannot characterize scRNA‐seq data. The high‐dimensional WGCNA (hdWGCNA) algorithm is an innovative approach that offers enhanced modularity for building co‐expression networks across various cellular and spatial levels, making it particularly suitable for scRNA‐seq data [22]. The scRNA‐seq data were analyzed using hdWGCNA version 0.2.18 [23]. Metacell objects were constructed by selecting genes expressed in at least 5% of cells. The scale‐free topology model was fitted to a soft power of eight after the fitting threshold was set to > 0.85. Gene expression profiles of co‐expressed modules can be represented by modular signature genes (MEs). To evaluate the correlation between modules and cell types, Spearman's test was used. PPI networks were constructed using HubGeneNetworkPlot.

### 2.9 Human Renal Glomerular Endothelial Cell Culture

We obtained human renal glomerular endothelial cells from BeNa Culture Collection (Beijing, China) and cultured them in accordance with our previous report [12]. Human RGECs were cultured in serum‐free medium for 12 h and then pretreated for 24 h with 100μmol/L Rac1 inhibitor NSC23766 (catalog no. 1177865‐17‐6, Sigma, St. Louis, MO, USA) before IL‐6 (100 ng/mL) combined with IL‐6R (1500 ng/mL) stimulation. The cells were cultured for 48 h, followed by collection and extraction of total protein for subsequent analysis.

### 2.10 Western Blot Analysis

As described previously [12], Western blot analysis was performed using primary antibodies against VE‐cad (1:500; catalog no. sc‐9989; Santa Cruz Biotechnology Inc., Dallas, TX, USA), Rac1 (1:1000; catalog no. 66122‐1‐Ig; Proteintech, Wuhan, China), and β‐actin (1:500; catalog no. WL01372; Wanleibio, Shenyang, China).

### 2.11 Pull‐Down Assay

In this study, Rac1 activity was measured using a pull‐down assay kit (catalog no. 8815; Cell Signaling Technology, Danvers, MA, USA) as directed by the manufacturer. A buffer containing 1% Triton X‐100, 20 mM HEPES, 150 mM NaCl, 5 mM MgCl2, and protease inhibitors was applied to lyse HRGECs (pH 7.4). Following centrifugation at 12,000 g for 10 min, total tissue protein was incubated with GST‐PBD at 4°C for 15 min. A buffer containing 0.01% NP40 was used to wash the pellets, boil them, and separate them by SDS‐PAGE. After that, Western blot analysis was performed.

## 3 Results

### 3.1 Definition of Clusters and Dimensionality Reduction Analysis

**Figure 1:**
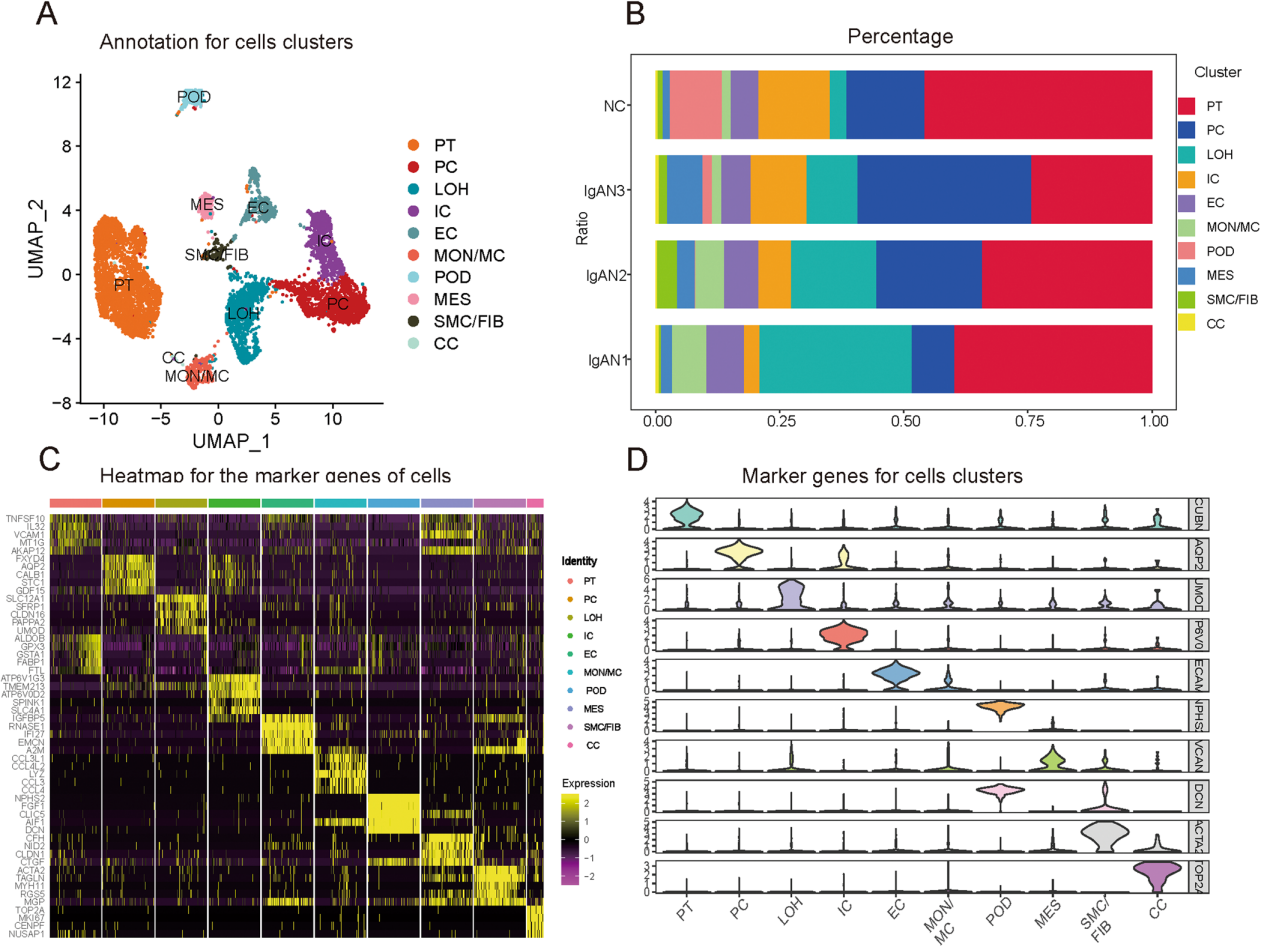
Cell clustering and annotation of scRNA‐seq data. (A) The 11 cell subpopulations were defined by UMAP cluster analysis and labeled according to different marker genes. (B) Frequency of each cell cluster in different kidney samples. (C) Heat mapping of the first 20 genes of each of the 11 cell clusters showed good differentiation between different cells and reliable cell clustering (Figure S1D). (D) Marker genes and their expression levels in different cells were used to create stacked violin maps. CC, circulating cells; EC, vascular endothelial cells; IC, intercalated cells; LOH, loop of Henle cells; MES, mesangial cells; MON/MC, monocytes/macrophages; PC, principal cells; POD, podocytes; PT, proximal tubule cells; scRNA‐seq, single‐cell RNA sequencing; SMC/FIB, smooth muscle cells/fibroblasts; UMAP, unified manifold approximation and projection.

There are certain kidney diseases that are cell type‐specific. To determine the major cell types involved in IgAN, bioinformatic analysis was performed on the scRNA‐seq data set GSE171314 from the NCBI database. Using the Percentage Feature Set function, 19,830 cells were filtered and analyzed from the scRNA‐seq data. IgAN sample GSM5222732, which contained only 673 active cells, was removed from the analysis. All remaining samples were evenly distributed in terms of mRNA, distinct molecular identifiers, mitochondrial content, and rRNA content (Figure S1A,B). The “subset” function was applied to selected cells, resulting in 7533 cells. Using the ScaleData function, all genes from GSE171314 were scaled, and dimensionality reduction was performed by principal component analysis (Figure S1C). Using UMAP cluster analysis, 11 cell clusters were identified. (Figure 1A) and information from the CellMarker database was utilized to tag and annotate the cells (Table S1). There were proximal tubule cells, principal cells, Henle cells loops, intercalated cells, mesangial cells, smooth muscle cells/fibroblasts, and circulating cells identified. For each kidney sample, Table S2 presents the number of cells in each cluster; Figure 1B shows the frequency of each cell cluster. Using heat maps of the top 20 marker genes, the cell classification method was demonstrated to be reliable (Figure 1C, Figure S1D); endothelial cells were identified as a critical cell population. Violin diagram (Figure 1D) depicts the expression of gene markers specific to different cell lines.

### 3.2 Differential Gene Enrichment Analysis and AUCell Analysis

**Figure 2:**
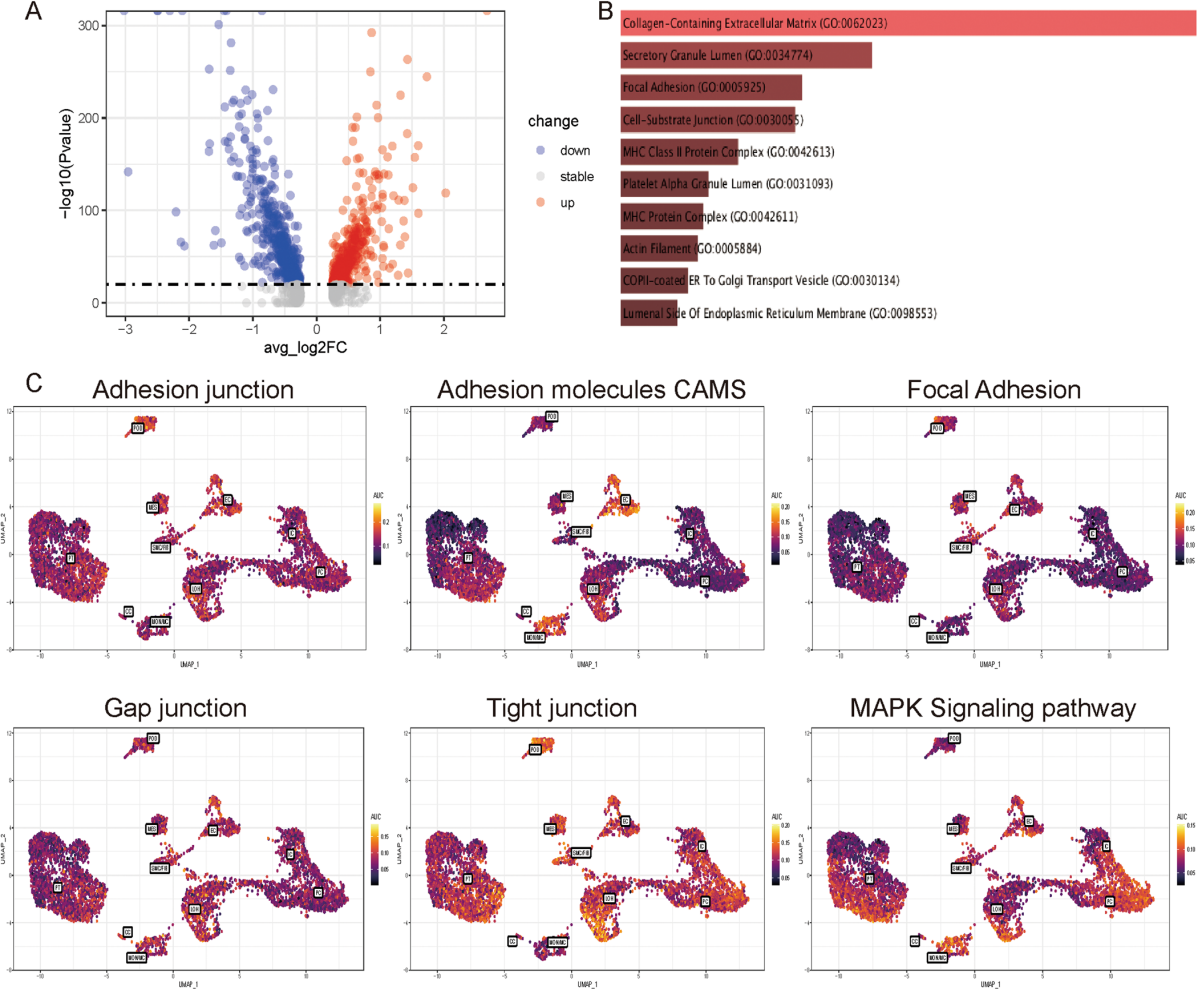
Differential gene AUCell analysis of IgAN and control kidney tissue. (A) Significant differences in gene expression were observed between the IgAN and control groups. (B) GO cellular component analysis identified focal adhesion as a pathway. (C) AUCell analysis showed that focal adhesion, CAM, and MAPK pathway‐related genes were significantly expressed in endothelial cells. CAM, cell adhesion molecule; GO, gene ontogeny; IgAN, IgA nephropathy; MAPK, mitogen‐activated protein kinase.

Figure 2A shows the DEGs between the IgAN and control groups (Supplementary Table 3). Enrichr (https://maayanlab.cloud/Enrichr/) was used to analyze the first 100 DEGs, and focal adhesion was identified as one of the pathways (Figure 2B). The expression of genes involved in adhesion, focal adhesion, cell adhesion molecules (CAMs), tight junctions, gap junctions, and the adhesion‐related mitogen‐activated protein kinase (MAPK) signaling pathway in different cell types was analyzed by AUCell (Figure 2C) to examine the role of adhesion. In AUCell analysis, CAMs and the MAPK signaling pathway (CAMs in particular) were more active in endothelial cells, suggesting that endothelial cells play a crucial role in IgAN pathogenesis.

### 3.3 Identification of Gene Expression Changes in Endothelial Cells

**Figure 3:**
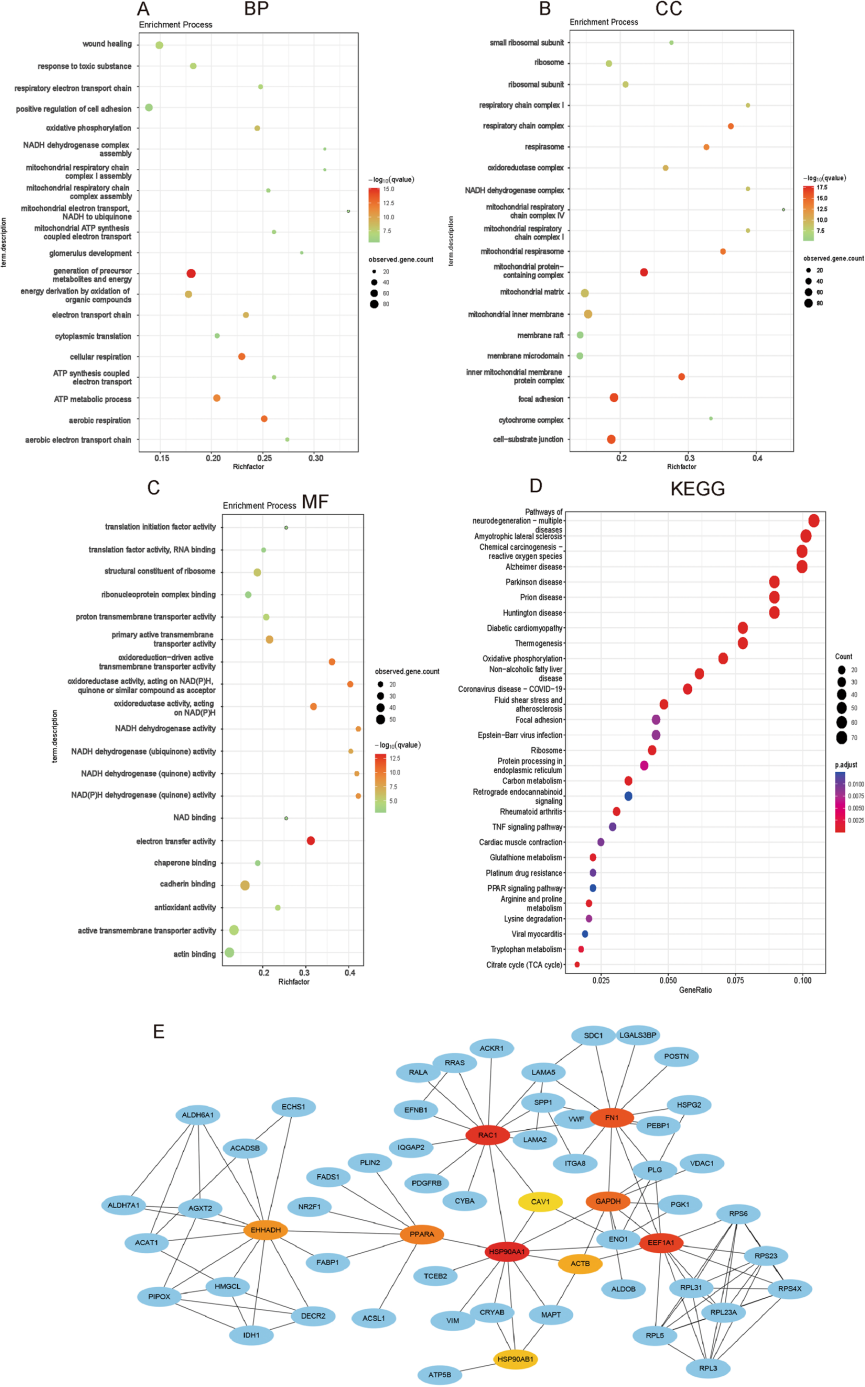
Differential gene enrichment analysis of renal endothelial cells from IgAN and control kidney tissues. (A) Cell adhesion was enriched in the BP of GO analysis. (B) Cell‐substrate junctions and focal adhesions were enriched in the CC of GO analysis. (C) Cadherin binding was further targeted in the MF analysis of GO analysis. (D) KEGG analysis showed enrichment of the focal adhesion pathway. (E) The hub genes HSP90AA1, RAC1, EEF1A1, FN1, GAPDH, PPARA, EHHADH, ACTB, HSP90AB1, and CAV1 were identified through CytoHubba analysis using the bottleneck method. BP, biological process; CC, cellular component; GO, gene ontogeny; IgAN, IgA nephropathy; KEGG, Kyoto Encyclopedia of Genes and Genomes; MF, molecular function.

To identify DEGs between IgAN and control groups, renal endothelial cells were selected from cell clusters (Table S4). To determine the function of these DEGs, KEGG and GO enrichment analyses were conducted. Based on the GO enrichment analysis, 1303 entries were enhanced by BP (*p* = 0.05), and Figure 3A illustrates the results of the first 20 annotations. Figure 3B shows the first 20 CC terms that are enhanced for focal adhesion. A total of 197 terms were enriched in MF; Figure 3C shows the top 20, including cadherin binding. Based on GO enrichment analysis, the DEGs were mainly associated with focal adhesions, cell‐substrate junctions, and cadherin binding. According to KEGG analysis, 92 pathways were identified (Figure 3D, *p* < 0.05), including the focal adhesion pathway.

### 3.4 Identification of Hub Genes

The top 500 DEGs between IgAN and control renal endothelial cells were entered into the STRING database to obtain 958 nodes, which were set to be > 0.9. Cytoscape data were imported, and hub genes were identified using CytoHubba and the bottleneck method. Hub genes (Figure 3E) included HSP90AA1, RAC1, EEF1A1, FN1, GAPDH, PPARA, EHHADH, ACTB, and HSP90AB1. A small GTPase called Rac1 plays a crucial role in intercellular adhesion by regulating the MAPK pathway [24].

### 3.5 Screening for Endothelial Cell‐Associated Modules Using hdWGCNA

**Figure 4:**
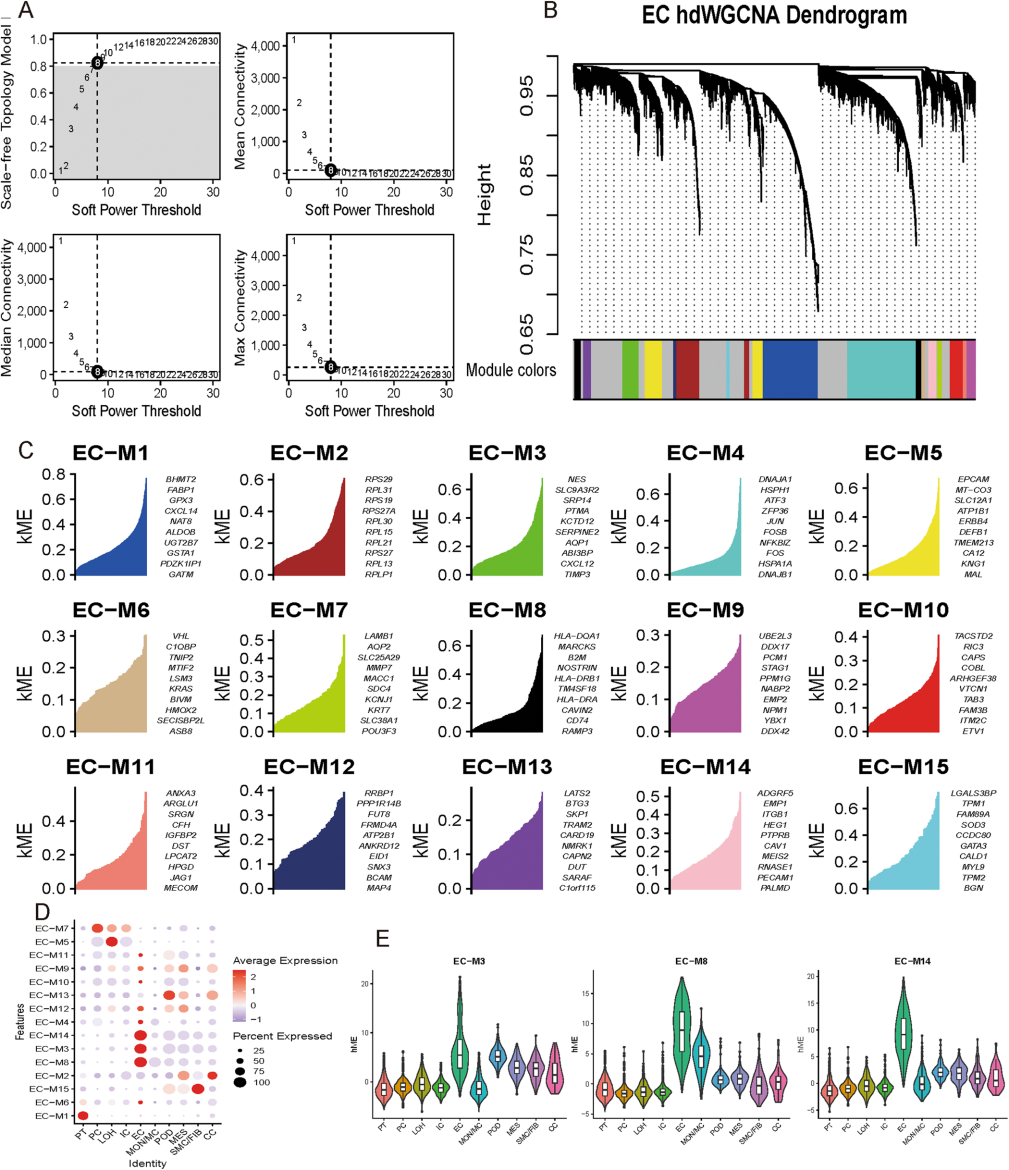
HdWGCNA of the hub modules strongly associated with renal endothelial cells. (A) Scale‐free networks were constructed using a soft power threshold of 8. (B) A dendrogram was created to visualize the 15 modules in the scale‐free network. (C) The top 10 hub genes of each module are shown; kME represents the correlation between this gene and the module. (D) Correlation analysis between modules and cells showed that renal endothelial cells were associated with modules 3, 8, and 14. (E) The expression of genes related to module 3 (green), module 8 (black), and module 14 (pink) in endothelial cells was significantly higher than that in other cells. HdWGCNA, high‐dimensional weighted co‐expression network analysis.

By using hdWGCNA, we deciphered the DEGs between IgAN and control renal endothelial cells in the scRNA‐seq data. A scale‐free topology model was constructed using a soft threshold of eight (Figure 4A). Except for the gray modules, the final analysis revealed 15 modules based on a scale‐free network architecture. Figure 4B shows a tree diagram of these modules. The hub genes for the different modules were further computed (Table S5). Our analysis of hub gene expression in different modules revealed significant differences between cells (Figure 4C). A ME represents the overall gene expression profile within each module and is used to infer the module's functional profile. Figure 4D shows the highest correlation between modules 3 (green), 8 (black), and 14 (pink) and renal endothelial cells. Module genes expressed distinctly in various cells also suggested that renal endothelial cells expressed genes related to modules 3, 8, and 14 separately from other cells (Figure 4E).

### 3.6 Hub Modules Gene Enrichment Analysis

**Figure 5:**
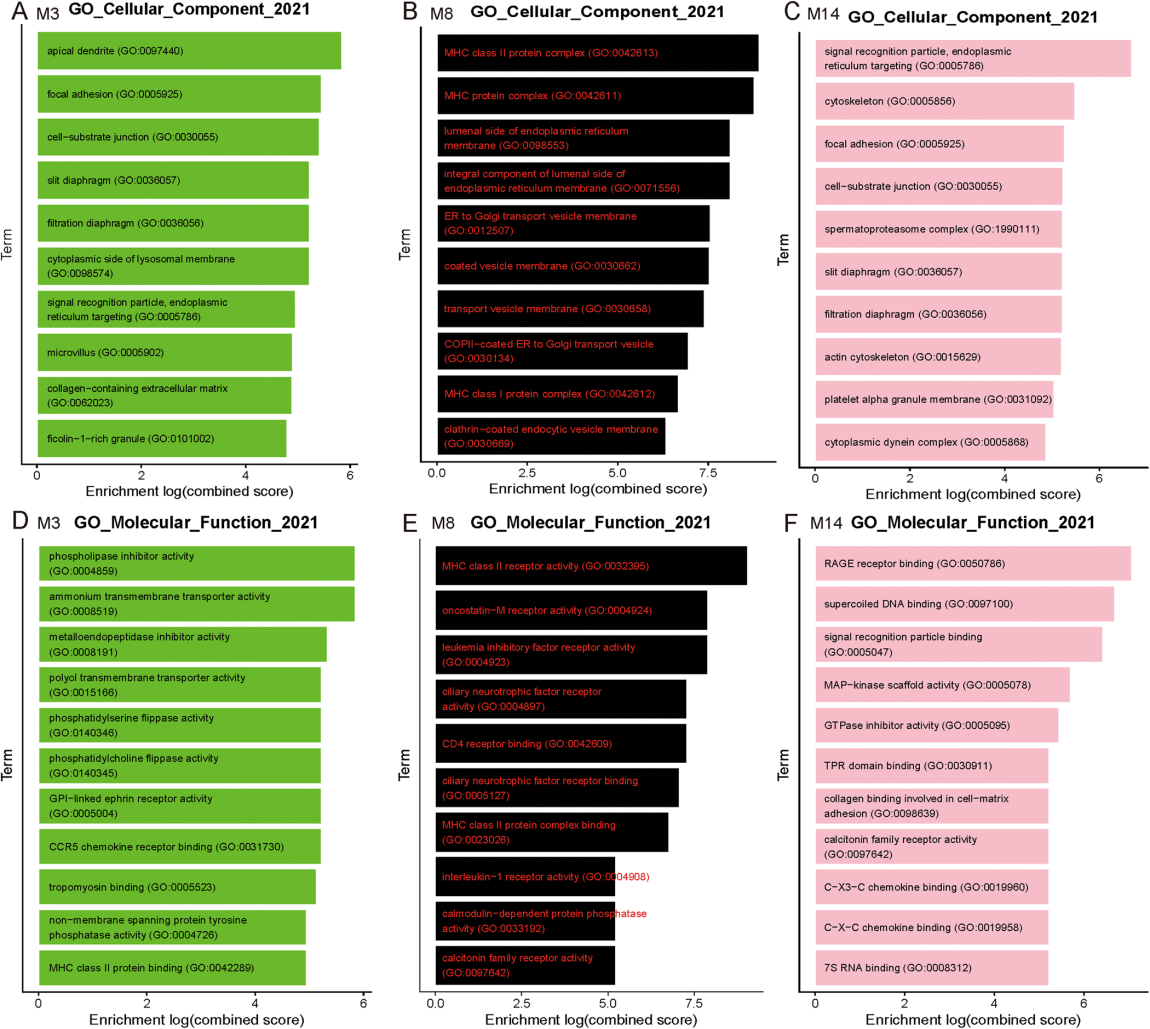
Hub gene enrichment analysis of endothelial cell‐related modules. (A) The module 3 hub gene analyzed by GO CC was enriched in focal adhesion, slit diaphragm, and filtration diaphragm. (B) GO CC enrichment analysis of the module 8 hub gene. (C) The module 14 hub gene analyzed by GO CC was enriched in focal adhesion, cytoskeleton, slit diaphragm, and filtration diaphragm. (D) The results of GO MF enrichment analysis of the module 3 hub gene. (E) The hub gene of module 8 was enriched by GO MF in leukemia inhibitory factor receptor activity. (F) The hub gene of module 14 analyzed by GO MF was enriched in MAPK and GTPase inhibitor activity. CC, cellular component; GO, gene ontogeny; MAPK, mitogen‐activated protein kinase; MF, molecular function.

An enrichment analysis was conducted on the top 100 hub genes of the related modules of renal endothelial cells. Based on GO CC enrichment analysis, modules 3 and 14 were enriched in focal adhesion, cytoskeleton, slit diaphragm, and filtration diaphragm (Figure 5A–C). Based on these results, modules 3 and 14 hub genes have a role in adhesion and hiatus membrane formation in renal endothelial cells. According to GO MF enrichment analysis (Figure 5D–F), module 8 hub genes exhibited leukemia inhibitory factor receptor activity, an interleukin‐6 family member. Genes related to IL‐6 were also included in the interaction diagram between the hub genes in module 8 (Figure S2). According to these results, IL‐6 is involved in renal endothelial cell injury. Module 14 hub genes enriched in MAPK and GTPase inhibitor activities were found by GO MF enrichment analysis (Figure 5F).

### 3.7 Effect of IL‐6 on HRGECs

**Figure 6:**
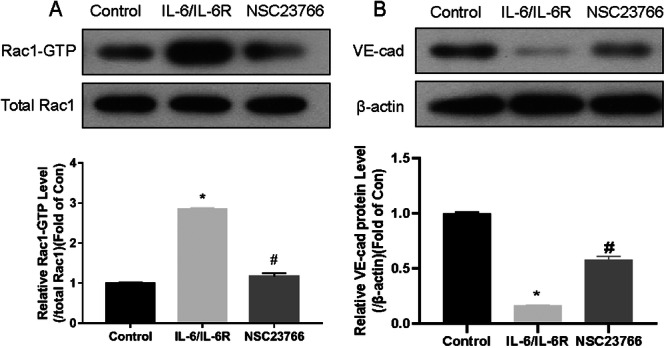
Effect of IL‐6 on VE‐cad expression in HRGECs. (A) IL‐6/IL‐6R had no effect on Rac1 expression in HRGECs, but increased Rac1‐GTP expression (*p* < 0.01). The Rac1 inhibitor NSC23766 reduced the increase in Rac1‐GTP expression (*p* < 0.01). (B) IL‐6/IL‐6R stimulation reduced the expression of VE‐cad in HRGECs (*p* < 0.01); NSC23766 ameliorated this effect (*p* < 0.01, *n* = 3). *p < 0.01, compared with the control; #*p* < 0.01, compared with the IL‐6/IL‐6R group; IL‐6, interleukin‐6; IL‐6R, interleukin‐6 receptor; HRGECs, human renal glomerular endothelial cells; VE‐cad, vascular endothelial cell cadherin.

Previously, we showed that IL‐6 decreased VE‐cad expression in HRGECs, increasing permeability and possibly contributing to IgAN endothelial cell lesions [12]. Rac1 plays a crucial role in the movement of various cells, including endothelial cells [25, 26], and may also be implicated in glomerular endothelial lesions. IL‐6 not only participates in the production of abnormal galactosylated IgA1 but also promotes the deposition of immune complexes [27]. IL‐6/IL‐6 receptor (IL‐6R) stimulation stimulated HRGECs. Rac1 inhibitor NSC23766, which blocks the Tiam‐1‐induced conversion of Rac1‐GDP to Rac1‐GTP [28], was then applied to the cells. The total Rac1 expression did not change following stimulation with IL‐6/IL‐6R in HRGECs (*p* > 0.05), but Rac1‐GTP expression was considerablyhigher in the IL‐6/IL‐6R group (*p* < 0.01). In Figure 6A, Rac1‐GTP expression decreased significantly after NSC23766 treatment (*p* < 0.01), demonstrating the inhibitor's efficacy. As shown in Figure 6B, treatment of HRGECs with IL‐6/IL‐6R significantly reduced VE‐cad expression (*p* < 0.05); this effect was alleviated by NSC23766 (*p* < 0.05). Based on these results, it appears that IL‐6/IL‐6R downregulates VE‐cad expression in HRGECs via Rac1.

## 4 Discussion

ScRNA‐seq allows gene expression analysis in multicellular systems at high resolution and depth. It is possible to miss rare cell types such as endothelial cells and podocytes when clustering single‐cell data from a single sample type. This study detected 484 endothelial cells based on a comprehensive analysis of 7533 cells to ensure data reliability. It can be challenging to generate scRNA‐seq data from adult kidney tissues due to rapid cell viability decline after tissue isolation [29]. As a control, cells with more than 40% mitochondrial gene expression were removed from the analysis. This was done along with sample GSM5222732, which had a low number of active cells. UMAP clustering analysis identified 11 different cell clusters (Figure 1A), and the labeling of these cells was mostly consistent with previous studies [13]. A heat map and stacked violin map of the clusters revealed significant differentiation between the cells (Figure 1C,D). The focal adhesion pathway was identified through enrichment analysis of DEGs from the IgAN and control groups (Figure 2B). CAMs and MAPK signaling pathway components were significantly expressed in endothelial cells according to AUCell analysis (Figure 2C). In addition to morphogenesis, immune responses, and thrombosis [30], CAMs participate in four broad categories of biological processes: integrins, cadherins, immunoglobulin superfamily members, and selectins. CAM expression is altered in glomerulonephritis [30] and transplant rejection [31]. There is some evidence that CAMs can act as biomarkers for systemic lupus erythematosus and rheumatoid arthritis [32], as well as reflect endothelial damage [33]. The MAPK signaling pathway is involved in cell proliferation, stress, inflammation, and differentiation, and in renal ischemia‐reperfusion injury [34] and mesangial cell proliferation in IgAN [35]. The findings of the present study (Figure 2C) are consistent with those of previous studies showing that CAM and MAPK are involved in endothelial damage in IgAN.

A further enrichment of renal endothelial cell DEGs identified focal adhesion, cell‐substrate junctions, and cadherin binding pathways (Figure 3A–D). IgAN also showed endothelial cell adhesion and junction injury. Through the MAPK signaling pathway, Rac1 regulates inflammation [24]. By impairing endothelial function, Rac1 and the MAPK pathway contribute to IgAN pathogenesis. Researchers have previously examined the role of renal endothelial cells in IgAN [36]; however, our findings demonstrate, for the first time, the involvement of CAMs in endothelial cell injury in IgAN (Figure 2C).

We used hdWGCNA to decipher gene expression differences in renal endothelial cells between IgAN and control kidney samples (Figure 4A–E). This was to investigate the intrinsic properties of our scRNA‐seq data. IL‐6, Rac1, and cadherin were hub genes linked to modules 3, 8, and 14, which were closely related to renal endothelial cells. In addition to affecting the trans‐signaling pathway that leads to vascular endothelial cell damage [37], IL‐6 also acts via Rac1 [38]. As previously reported, IL‐6 affects glomerular vascular endothelial cell permeability through VE‐cad [12]. A variety of chronic kidney disease models exhibit overactive Rac1, and Rac1 inhibitors protect kidneys [39]. Adhesion junctions and focal adhesions are complex macromolecular protein assemblies situated within the cell membrane at the interfaces of cell–cell and cell–matrix interactions, respectively. The interaction between two connections can be influenced by alterations in the tension of the actin network, resulting in cross‐talk [40]. The involvement of VE‐cad in the connection between cells of Adhesion junctions and the impact of integrins on the strength of focal adhesion‐extracellular matrix connection through actin remodeling during VE‐cad adhesion were investigated [41]. Therefore, our focus was to determine whether IL‐6 influences the expression of VE‐cad in glomerular endothelial cells?

IL‐6's role in endothelial cells was further investigated by treating HRGECs with IL‐6/IL‐6R. NSC23766 ameliorated VE‐cad downregulation by inhibiting Rac1. Thus, IL‐6 regulates VE‐cad expression through the pro‐inflammatory effector Rac1. There has been a significant increase in serum VE‐cad levels in sepsis, indicating damage to adhesion junctions between vascular endothelial cells [9]. Furthermore, IL‐6 has been shown to increase umbilical vein endothelial cells' permeability by decreasing VE‐cad expression, which is consistent with our findings (Figure 6) [42].

IL‐6 receptor antagonists alleviate Castleman disease which associated with IgA nephropathy [43]. Our previous studies demonstrated the role of IL‐6 in an animal model of IgA nephropathy [12]. This study further validated the significant role of IL‐6 on intercellular connectivity in IgAN in human renal glomerular endothelial cells. Several limitations of this study should be noted. Technical limitations of ScRNA‐seq include biases in transcript coverage, sequencing coverage, and low capture efficiency. As a result of these limitations, scRNA‐seq data have a higher degree of noise than bulk RNA‐seq data [28]. Even so, single‐cell omics is rapidly evolving, and their integration into multiomics assays will undoubtedly provide new insight into glomerular disease pathophysiology.

## 5 Conclusions

We report here for the first time the aberrant expression of important molecules implicated in adhesion junctions between renal endothelial cells in IgAN using scRNA‐seq data from human kidney tissue. IL‐6 downregulates VE‐cad expression in endothelial cells through Rac1 and may contribute to IgAN pathogenesis. Our results indicate that renal endothelial cells play a significant role in IgAN pathogenesis. The findings may lead to a better understanding of IgAN's molecular mechanisms and suggest new therapeutic targets.

## Author Contributions

Conceptualization: Cheng‐Guang Zhao and Ping Zhou. Methodology: Yong‐Chang Yang. Software: Lin Zhu and Xiao‐Meng Wang. Validation: Jing‐Ying Zhao and Bo Zhang. Formal analysis: Jing‐Ying Zhao. Resources, Bo Zhang. Data curation: Bo Zhang. Writing – original draft preparation: Yong‐Chang Yang. Writing – review and editing: Cheng‐Guang Zhao and Wai W. Cheung. Supervision: Wai W. Cheung. Funding acquisition: Ping Zhou. All authors have read and agreed to the published version of the manuscript.

## Ethics Statement

The authors have nothing to report.

## Consent

The authors have nothing to report.

## Conflicts of Interest

The authors declare no conflicts of interest.

## Supporting information

Supporting information.

Supporting information.

Supporting information.

Supporting information.

## Data Availability

Publicly available datasets were analyzed in this study. These data are available at https://www.ncbi.nlm.nih.gov/geo/ (GSE171314). Additional data presented in this study are available on request from the corresponding authors.
