# Supplementary figures and images for: Single‐Cell Analysis of Endothelial Cell Injury in IgA Nephropathy
Source: Immun Inflamm Dis. 2025 Feb 13;13(2):e70149. doi: 10.1002/iid3.70149 (PMC11822453; doi:10.1002/iid3.70149)

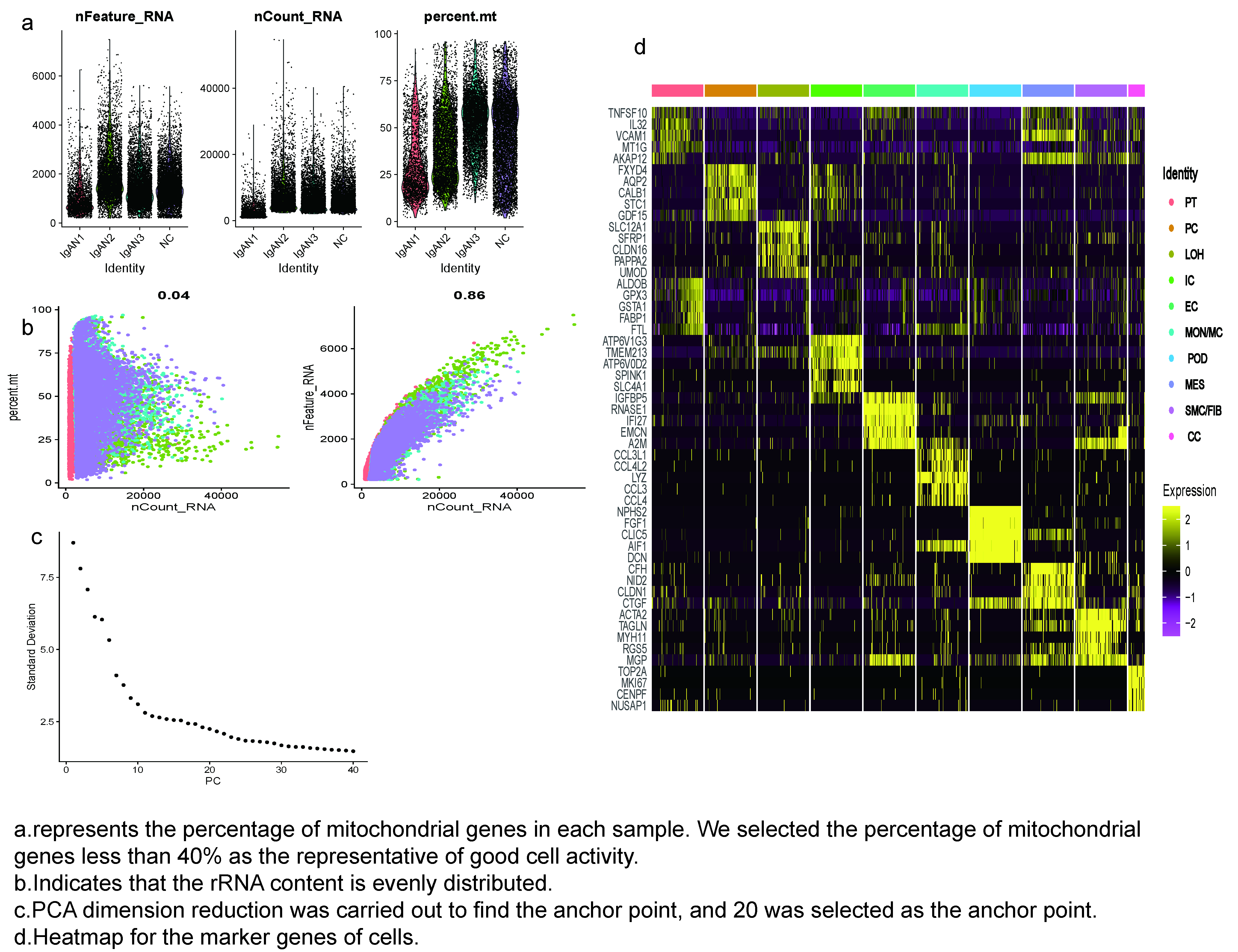

Supplement: Supplementary file 1 — Supporting information. [file IID3-13-e70149-s001.tif]

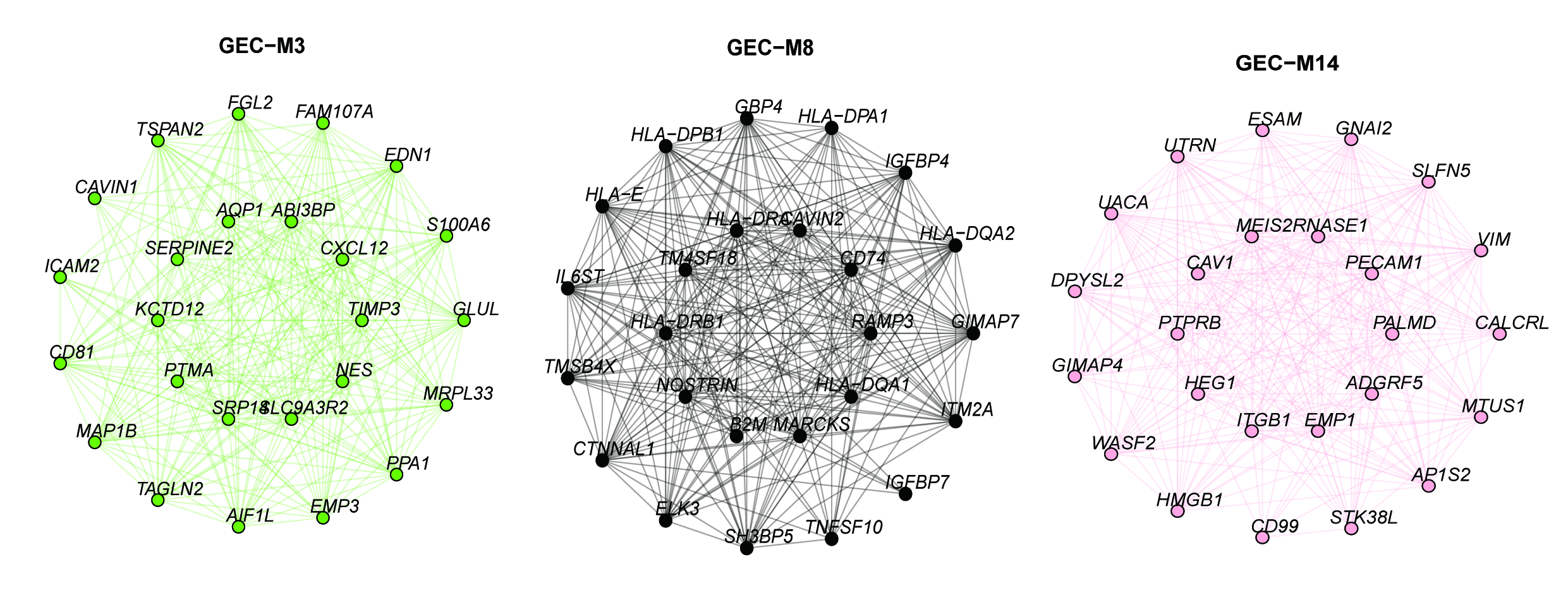

Supplement: Supplementary file 2 — Supporting information. [file IID3-13-e70149-s003.tif]
