# Supplementary material for: Single‐Cell Analysis of Endothelial Cell Injury in IgA Nephropathy
Source: Immun Inflamm Dis. 2025 Feb 13;13(2):e70149. doi: 10.1002/iid3.70149 (PMC11822453; doi:10.1002/iid3.70149)
Supplement: Supplementary file 3 — Supporting information. [file IID3-13-e70149-s004.doc]

| Cell type | Marker gene |
| --- | --- |
| Proximal tubular cells (PT) | LRP2, CUBN, SLC13A1, ALDOB |
| Principal cells (PC) | AQP2, AQP3 |
| Loop of Henle cells (LOH) | SLC12A1, CLDN16, UMOD |
| Intercalated cells (IC) | ATP6V1G3, SLC4A1, SLC26A7 |
| Endothelial cells (EC) | PECAM1, ACKR1, VWF, CLDN5 |
| Macrophages (MC)/Monocytes (MON) | LYZ, C1QB, C1QA, MRC1, FCN1, CD14, CD68 |
| Podocytes (POD) | NPHS2, PODXL, PTPRO |
| Mesangial cells (MES) | CTGF, FHL2, MYL9, FN1 |
| Smooth muscle cells (SMC)/Fibroblasts (FIB) | ACTA2, TAGLN, MYH11, DCN,MYLK, LUM |
| Cycling cells (CC) | TOP2A, MKI67 |

Supplementary Table 1. Cell-lineage-specific marker genes of different cell types.

Markers were made based on published kidney single cell related studies [43] and CellMarker database.

Supplementary Table 2. The number of different cells in each sample.

|  | IgAN1 | IgAN2 | IgAN3 | NC |
| --- | --- | --- | --- | --- |
| PT | 553 | 867 | 275 | 1143 |
| PC | 119 | 538 | 396 | 391 |
| LOH | 425 | 432 | 117 | 82 |
| IC | 44 | 166 | 127 | 358 |
| EC | 104 | 175 | 67 | 138 |
| MON/MC | 97 | 147 | 21 | 47 |
| POD | 0 | 3 | 22 | 259 |
| MES | 30 | 91 | 80 | 37 |
| SMC/FIB | 6 | 101 | 19 | 24 |
| CC | 9 | 6 | 7 | 10 |

A total of four renal biopsy specimens were collected from patients with IgAN (n = 3), and healthy control (n = 1) obtained from living donor kidney. All three IgAN patients had proteinuria ranged from 0.27 to 2.57 g per 24 h.

PT, proximal tubule cells; PC, principal cells; LOH, loop of Henle cells; IC, intercalated cells; EC, vascular endothelial cells; MON/MC, monocytes/macrophages; POD, podocytes; MES, mesangial cells; SMC/FIB, smooth muscle cells/fibroblasts; CC, circulating cells.
